# MR Corge: sensitivity analysis of Mendelian randomization based on the core gene hypothesis for polygenic exposures

**DOI:** 10.1093/bioinformatics/btae666

**Published:** 2024-11-08

**Authors:** Wenmin Zhang, Chen-Yang Su, Satoshi Yoshiji, Tianyuan Lu

**Affiliations:** Montreal Heart Institute, Montreal, QC, H1T 1C8, Canada; Quantitative Life Sciences Program, McGill University, Montreal, QC, H3A 0G4, Canada; Victor Phillip Dahdaleh Institute of Genomic Medicine, McGill University, Montreal, QC, H3A 0G1, Canada; Victor Phillip Dahdaleh Institute of Genomic Medicine, McGill University, Montreal, QC, H3A 0G1, Canada; Department of Human Genetics, McGill University, Montreal, QC, H3A 0G1, Canada; Lady Davis Institute for Medical Research, Montreal, QC, H3T 1E2, Canada; Programs in Metabolism and Medical & Population Genetics, The Broad Institute of MIT and Harvard, Cambridge, MA, 02142, United States; Harvard Medical School, Boston, MA, 02115, United States; Department of Population Health Sciences, University of Wisconsin-Madison, Madison, WI, 53726, United States; Department of Biostatistics and Medical Informatics, University of Wisconsin-Madison, Madison, WI, 53726, United States; Center for Demography of Health and Aging, University of Wisconsin-Madison, Madison, WI, 53706, United States; Center for Genomic Science Innovation, University of Wisconsin-Madison, Madison, WI, 53706, United States; Center for Human Genomics and Precision Medicine, University of Wisconsin-Madison, Madison, WI, 53705, United States

## Abstract

**Summary:**

Mendelian randomization is being utilized to assess causal effects of polygenic exposures, where many genetic instruments are subject to horizontal pleiotropy. Existing methods for detecting and correcting for horizontal pleiotropy have important assumptions that may not be fulfilled. Built upon the core gene hypothesis, we developed MR Corge for performing sensitivity analysis of Mendelian randomization. MR Corge identifies a small number of putative core instruments that are more likely to affect genes with a direct biological role in an exposure and obtains causal effect estimates based on these instruments, thereby reducing the risk of horizontal pleiotropy. Using positive and negative controls, we demonstrated that MR Corge estimates aligned with established biomedical knowledge and the results of randomized controlled trials. MR Corge may be widely applied to investigate polygenic exposure-outcome relationships.

**Availability and implementation:**

An open-sourced R package is available at https://github.com/zhwm/MRCorge.

## 1 Introduction

Mendelian randomization (MR) is a promising tool for assessing potential causal relationships (Davey Smith and Ebrahim 2003, Smith and Ebrahim 2004) since randomized controlled trials are often limited by ethical and practical constraints. Using genetic variants as instrumental variables, MR may effectively mitigate the risks of confounding and reverse causation that frequently bias observational association studies (Davey Smith and Ebrahim 2003, Smith and Ebrahim 2004). MR relies on essential instrumental variable assumptions of relevance, independence, and exclusion restriction (Skrivankova *et al.* 2021a,b). Specifically, genetic instruments must be strongly associated with the exposure, be independent of any confounders, and affect the outcome only through the exposure. The relevance assumption can be fulfilled by selecting genetic variants demonstrating genome-wide significance in genome-wide association studies (GWAS) of exposures, while the independence assumption may be examined by testing for instrument-confounder associations. However, exclusion restriction, also known as the assumption of no horizontal pleiotropy, remains challenging to verify.

With the goal to robustly derive MR estimates in the presence of horizontal pleiotropy, several methods have been developed beyond the original Wald ratio and inverse variance weighted (IVW) methods (Burgess *et al.* 2013). For instance, the median-based methods (e.g. simple median, weighted median, and penalized weighted median methods) require that at least 50% of the instruments or their weights are valid (Bowden *et al.* 2016, Burgess *et al.* 2017). The mode-based methods (e.g. simple mode and weighted mode methods) require that the mode of the causal effect estimates or their weights is based on valid instruments (Hartwig *et al.* 2017). The MR PRESSO method identifies and removes outlier instruments (Verbanck *et al.* 2018), while the contamination mixture method identifies one or more groups of instruments with consistent causal estimates (Burgess *et al.* 2020), both having the assumption that a plurality of the instruments should be valid (Verbanck *et al.* 2018, Burgess *et al.* 2020). The MR Egger method and MR RAPS method allow for pervasive pleiotropic effects but have an additional assumption that the strength of the genetic instruments is independent of their direct effects on the outcome (i.e. InSIDE assumption) (Burgess and Thompson 2017, Zhao *et al.* 2020).

In recent years, MR has been increasingly utilized to estimate the potential causal effects of polygenic exposures, for which tens or hundreds of independent genetic variants can be identified as candidate instruments from GWAS of rapidly growing sample sizes (Visscher *et al.* 2017, Abdellaoui *et al.* 2023). It has been recognized that many, sometimes the vast majority, of these candidate instruments do not have a biologically interpretable role in the exposures and can be involved in multiple distinct biological pathways (Boyle *et al.* 2017, Liu *et al.* 2019b, Sinnott-Armstrong *et al.* 2021a). This substantially increases the risk of violating the additional assumptions of the aforementioned MR methods.

The core gene hypothesis in the theory of the omnigenic model (Boyle *et al.* 2017) has been proposed to explain the high polygenicity of complex traits. The core gene hypothesis states that genetic variants within core genes or pathways have more direct and specific effects on a polygenic trait than those within peripheral genes or pathways, which more likely play regulatory roles. We hypothesize that for polygenic exposures, using instruments from core genes or pathways may reduce the risk of horizontal pleiotropy.

In this work, we present MR Corge for performing sensitivity analysis of MR based on the core gene hypothesis. MR Corge estimates the effect of a polygenic exposure using a group of putative core instruments. We demonstrate the utility of MR Corge in assessing polygenic exposure-outcome relationships using positive and negative controls.

## 2 Methods

MR Corge relies on two important assumptions derived from the core gene hypothesis (Boyle *et al.* 2017). First, for a polygenic exposure, a small number of core genes have a direct biological impact on the trait. Second, genetic instruments with a stronger variant-trait association (i.e. core instruments) are more likely to act through core genes, whereas those with a weaker variant-trait association (i.e. peripheral instruments) are more likely to act through peripheral genes and be subject to horizontal pleiotropy. With these assumptions, MR estimates obtained based on the putative core instruments are less likely to be biased by horizontal pleiotropy than those based on all instruments.

In MR Corge, the most crucial step is to identify putative core instruments from all candidate instruments, which are typically pre-selected through linkage disequilibrium (LD) clumping based on GWAS summary statistics. Four instrument ranking options have been made available, based on

1.
$\left|{\hat{\beta }}_{j}\right|$ (default setting): the per-allele effect of the jth genetic variant on the exposure, estimated in the GWAS of the exposure;
2.
$f_{j}\left(1-f_{j}\right){\hat{\beta }}_{j}^{2}$ : the per-variant heritability of the jth genetic variant, which is proportional to the squared per-allele effect normalized by the minor allele frequency ($f_{j}$);
3.
$\frac{\left|{\hat{\beta }}_{j}\right|}{SE_{j}}$ : the significance of the jth genetic variant in the GWAS of the exposure;
4.
$\frac{\left|{\hat{\beta }}_{j}\right|}{SE_{j}\sqrt{N_{j}}}$ : the sample size ($N_{j}$)-normalized significance of the jth genetic variant, which can differ from (3) especially in meta-analyses of GWAS with multiple participating studies.

All pre-selected candidate instruments are ranked based on one of these four metrics in descending order. Among the tied instruments, ranks are randomly assigned. Then, the instruments are categorized into K groups, where each group contains approximately the same number of instruments. To showcase the utility of MR Corge, we choose K such that each group contains 5 to 10 instruments in this study. In practice, multiple sensitivity analyses should be conducted using different values of K.

**Figure 1.**
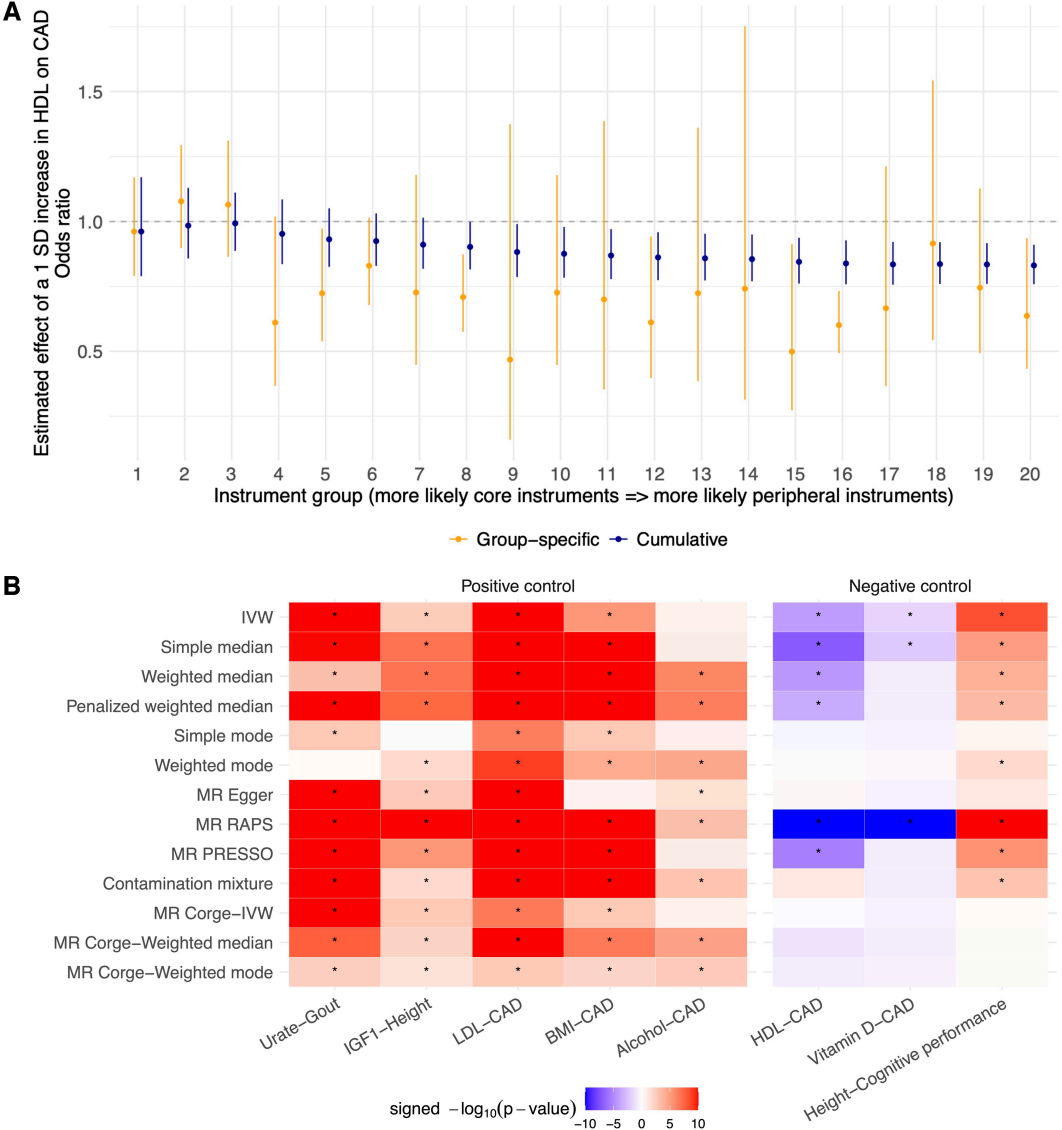
Benchmarking the performance of MR Corge. (A) Group-specific and cumulative MR Corge estimates of the effect of serum high-density lipoprotein cholesterol (HDL) levels on the risk of coronary artery disease (CAD). Estimates obtained using the inverse variance weighted method are illustrated with error bars indicating 95% confidence intervals. SD, standard deviation. (B) Significance of association estimates in positive and negative controls. Association estimates obtained with nonspecific negative control outcomes are presented in Supplementary Fig. S1. Asterisks (*) indicate estimates with a *P*-value < 0.05. *P*-values < 1.0 × 10^−10^ were set to 1.0 × 10^−10^ for visualization purposes. Positive values of signed significance represent that increased exposure was predicted to increase the outcome while negative values of signed significance represent that increased exposure was predicted to decrease the outcome. Instruments were ranked based on the default ranking criterion based on the absolute value of per-allele effect size.

MR Corge then generates group-specific and cumulative MR estimates that can be based on any existing methods (Fig. 1A). By default, MR Corge applies the IVW, weighted median, and weighted mode methods for estimation. The kth cumulative MR estimate (k∈{1,…,K}) is obtained based on all instruments in the kth and all preceding groups, if any. An F-statistic is calculated for each group of instruments to assess whether the MR estimates may be subject to weak instrument bias (Burgess *et al.* 2011). MR estimates based on the first group of instruments, which are putative core instruments, are the primary MR Corge estimates.

We utilized several positive and negative controls to examine whether sensible MR estimates could be obtained using MR Corge.

Positive controls:

1. Serum insulin-like growth factor 1 (IGF1)-height association: IGF1 mediates the effects of growth hormone in various tissues, promotes the proliferation and differentiation of chondrocytes and myoblasts, and supports energy production, resulting in bone elongation and height increase (Martin and Baxter 1986, Clemmons 2018, Yakar *et al.* 2018);
2. Serum urate-gout association: Gout is primarily caused by elevated levels of circulating urate, which can form urate crystals in joints and trigger inflammatory responses (Dalbeth *et al.* 2019);
3. Serum low-density lipoprotein cholesterol (LDL)-coronary artery disease (CAD), body mass index (BMI)-CAD, and alcohol intake-CAD associations: The risk-increasing effects of hyperlipidemia, obesity, and excessive alcohol intake on CAD have been confirmed in previous randomized controlled trials as well as Mendelian randomization and observational studies (Wilson 1994, Dansinger *et al.* 2005, Baigent *et al.* 2010, Sabatine 2019, Biddinger *et al.* 2022).

Negative controls:

1. Serum high-density lipoprotein cholesterol (HDL)-CAD and vitamin D-CAD associations: Although increased HDL and vitamin D levels have been associated with a decreased risk of CAD in observational studies, randomized controlled trials have shown no meaningful protective effects of HDL-increasing drugs or vitamin D supplements against CAD in the general population with normal HDL or vitamin D levels (Nissen *et al.* 2007, Lincoff *et al.* 2017, Scragg *et al.* 2017, Manson *et al.* 2019);
2. Height-cognitive performance association: Height is a significant predictor of cognitive performance in observational studies (Tanner 1966), yet this association can be strongly confounded by various factors, such as socioeconomic status, nutrition, and assortative mating. We assessed the height-cognitive performance association using LD score regression (version 1.0.1) (Bulik-Sullivan *et al.* 2015a,b) to estimate the genetic correlation based on within-sibship studies, which can effectively control for unmeasured confounders (Howe *et al.* 2022).
3. Following previous work (Sanderson *et al.* 2021, Hu *et al.* 2024), we also used natural hair color and self-reported ease of skin tanning(Watanabe *et al.* 2019) as nonspecific negative control outcomes, with IGF1, urate, LDL, BMI, HDL, vitamin D, and height as the exposures.

GWAS summary statistics of these exposures and outcomes were obtained from large-scale studies (Supplementary Table S1) (Köttgen *et al.* 2013, Willer *et al.* 2013, Wood *et al.* 2014, Locke *et al.* 2015, Lee *et al.* 2018, Liu *et al.* 2019a, Manousaki *et al.* 2020, Sinnott-Armstrong *et al.* 2021b, Aragam *et al.* 2022). For each exposure, we performed LD clumping using the ieugwasr R package (version 1.0.1) (Hemani *et al.* 2018) with an LD reference panel generated from 50 000 randomly sampled UK Biobank participants of European ancestry (Bycroft *et al.* 2018). We retained independent genetic variants (LD *r*^2^ < 0.001, window size = 1 Mb) that demonstrated genome-wide significance (*P*-value < 5.0×10^−8^) as candidate instruments. The summary statistics of these instruments were obtained from the GWAS of the corresponding outcomes. When an instrument was not present, we attempted to identify its proxy variant with an LD *r*^2^ > 0.8 using the same LD reference panel. Data harmonization was performed using the TwoSampleMR R package (version 0.5.7) (Hemani *et al.* 2018). Palindromic variants a minor allele frequency > 0.42 were excluded. MR Corge estimates were obtained using the IVW, weighted median, and weighted mode methods, with K = 5 for the alcohol intake, K = 20 for IGF1, urate, LDL, BMI, HDL, and vitamin D, and K = 100 for height, where each instrument group contains 5–10 instruments. All genetic instruments and their functional impacts on nearby genes annotated using the Ensembl Variant Effect Predictor (release 112) (McLaren *et al.* 2016) are detailed in Supplementary Table S2.

We also derived MR estimates for each of these exposure-outcome pairs based on all selected instruments throughout the genome, using the IVW, simple median, weighted median, simple mode, weighted mode, penalized weighted median, MR Egger, MR RAPS, MR PRESSO, and contamination mixture methods. All MR estimates are provided in Supplementary Tables S3 and S4.

## 3 Results

### 3.1 Positive controls

Using the default instrument ranking criterion based on the absolute value of per-allele effect size, the putative core instrument groups contained genetic variants mapping to genes with well-established biological functions related to the exposures. For instance, the putative core instruments for serum IGF1 levels included rs856540, an intergenic variant near *IGFBP3* (main carrier of IGF1), while the putative core instruments for serum urate levels included rs1165196, a missense variant of *SLC17A1*, and rs1171614, an intronic variant of *SLC16A9*, where both SLC17A1 and SLC16A9 are important urate solute carriers. MR Corge estimates consistently showed that increased serum IGF1 levels could lead to increased height while increased serum urate levels could elevate the risk of gout (Fig. 1B). Meanwhile, serum LDL levels and BMI were predicted to increase the risk of CAD (Fig. 1B). Without selecting instruments, the simple mode, weighted mode, and MR Egger methods did not identify all of these associations (Fig. 1B).

The strongest instrument for alcohol intake was rs1229984, a missense variant of *ADH1B* (alcohol dehydrogenase). ADH1B regulates the accumulation of acetaldehyde which can cause unpleasant effects such as flushing, nausea, and rapid heart rate (Polimanti and Gelernter 2018, Lu *et al.* 2023a,b). However, most of the other instruments do not have a direct role in the metabolism of alcohol. With the core instruments, MR Corge estimates obtained using the weighted median and weighted mode methods identified the risk-increasing effect of alcohol intake on CAD, although the MR Corge estimate using the IVW method, along with four other methods based on all instruments, did not identify this effect (Fig. 1B).

### 3.2 Negative controls

Furthermore, MR Corge estimates consistently showed that serum HDL and vitamin D levels may not be causally associated with CAD, while based on all instruments, the IVW, simple median, weighted median, penalized weighted median, MR RAPS, and MR PRESSO suggested protective effects of at least one exposure (Fig. 1B). The putative core instruments for HDL levels included rs9989419, an intergenic variant near *CETP* [cholesteryl ester transfer protein, transferring cholesterol esters from HDL particles to other lipoproteins (Chapman *et al.* 2010)], while the putative core instruments for vitamin D levels included rs117576073, an intronic variant of *CYP2R1* (vitamin D 25-hydroxylase), rs4694423, an intergenic variant near *GC* (vitamin D binding protein), and rs12803256, an intergenic variant near *DHCR7* [7-dehydrocholesterol reductase which balances biosynthesis of cholesterol and vitamin D (Prabhu *et al.* 2016)]. Interestingly, consistent with MR Corge estimates, CETP-inhibitors have been a recent focus of drug development yet have not shown effectiveness in reducing cardiovascular disease risks in randomized controlled trials, despite their capacity to significantly elevate serum HDL levels (Nissen *et al.* 2007, Lincoff *et al.* 2017).

Meanwhile, MR Corge estimates indicated that height was not associated with cognitive performance, consistent with the absence of genetic correlation based on within-sibship studies (Supplementary Table S5). In contrast, when all instruments were utilized, eight of the ten MR methods suggested a causal effect of increased height on increased cognitive performance (Fig. 1B).

In addition, MR Corge demonstrated appropriate false positive control with natural hair color and self-reported ease of skin tanning as nonspecific negative control outcomes (Supplementary Fig. S1).

## 4 Discussion

Instrumental variable assumptions are essential for deriving valid MR estimates. It has been recognized that carefully selecting genetic instruments which represent relevant biological mechanisms may mitigate the risk of horizontal pleiotropy, such as using lead variants in cis-quantitative trait loci for instrumenting the abundances of mRNA transcripts, proteins, and certain metabolites (Porcu *et al.* 2019, Zhou *et al.* 2021, Chen *et al.* 2023, Lu *et al.* 2023a,b, Yoshiji *et al.* 2023, Lu *et al.* 2024). However, assessing potential causal effects of many polygenic exposures using MR remains challenging since the underlying biological mechanisms may not have been fully elucidated and a large number of candidate instruments can be subject to horizontal pleiotropy. MR Corge can be a useful tool for performing sensitivity analysis for such exposures.

With positive and negative controls, we demonstrated that MR estimates based on all instruments could be inconsistent with established biomedical knowledge or the results of randomized controlled trials, even when robust MR methods were used. This inconsistency is likely due to the violation of additional assumptions of these methods. In contrast, with a small number of putative core instruments, the assumptions of robust MR methods, such as the weighted median and weighted mode methods, are more likely to be fulfilled due to the enrichment of valid instruments. As a result, MR Corge could consistently provide sensible MR estimates. Moreover, because the putative core instruments had the strongest associations with the exposures, the F-statistics of the core instrument groups were higher than the overall F-statistics based on all instruments (Supplementary Table S4), indicating a low risk of introducing weak instrument bias (Burgess *et al.* 2011) and further supporting that appropriate instrument selection should prevail over maximizing the proportion of exposure variance explained by the instruments. This principle likely also applies to analyses using genetic risk scores as instrumental variables, where existing methods for addressing horizontal pleiotropy are not applicable.

Similar to other methods that operate on GWAS summary statistics, MR Corge may yield biased results if the GWAS populations are subject to selection bias (Pirastu *et al.* 2021) or if the genetic effect estimates capture the effects of demography or relatives (Howe *et al.* 2022). Identifying data sources less likely to introduce such bias remains crucial for analyses using MR Corge.

Notably, the choice of K directly reflects the fundamental assumption regarding the number of core instruments, where a larger K corresponds to sparser core instruments and core genes. In practice, given the significant knowledge gap between the genetic architecture and biological mechanisms of most polygenic traits, a universal rule for determining core instruments may not exist, and the core instrument-peripheral instrument classification may not be binary (Boyle *et al.* 2017). We recommend conducting multiple sensitivity analyses with different values of K (Supplementary Fig. S2), where consistent results are more likely to support causal interpretations. Furthermore, empirically, we found that ranking based on the absolute value of per-allele effect size outperformed other ranking criteria (Supplementary Fig. S3). However, interpreting MR Corge estimates always requires cautious investigation of the putative core instruments, as well as triangulation of multiple lines of evidence, as with other MR or epidemiological methods.

In summary, built upon the core gene hypothesis, MR Corge may identify putative core instruments to obtain MR estimates that are less likely to be biased by horizontal pleiotropy. MR Corge may have wide utility as a tool for performing sensitivity analysis when the exposures demonstrate high polygenicity, while the underlying biological mechanisms remain unclear.

## Supplementary Material

btae666_Supplementary_Data
