# Supplementary material for: MR Corge: sensitivity analysis of Mendelian randomization based on the core gene hypothesis for polygenic exposures
Source: Bioinformatics. 2024 Nov 8;40(11):btae666. doi: 10.1093/bioinformatics/btae666 (PMC11578597; doi:10.1093/bioinformatics/btae666)
Supplement: btae666_Supplementary_Data [file btae666_supplementary_data.zip › MRCORGE.supp.final.pdf]

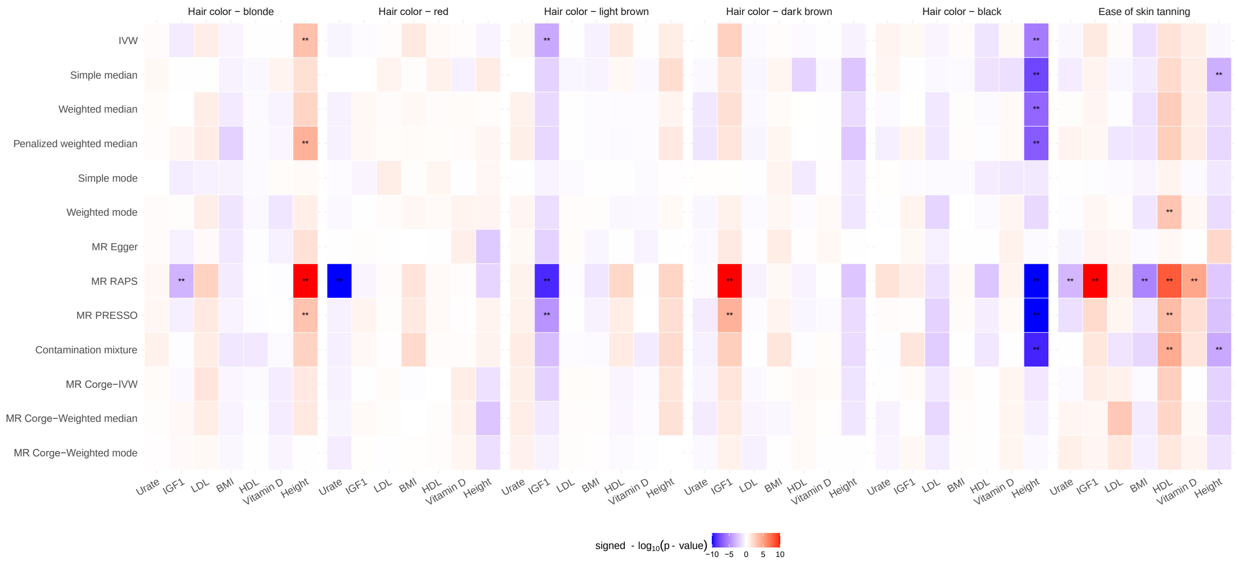

Supplementary Figure S1. Significance of association estimates with non-specific negative control outcomes. Double asterisks (**) indicate estimates with a p-value < 1.2×10^{-3}, which is the Bonferroni-corrected significance threshold accounting for 42 tests (7 exposures and 6 outcomes). P-values < 1.0×10^{-10} were set to 1.0×10^{-10} for visualization purposes. Red colors represent that increased exposure was predicted to increase the outcome while blue colors represent that increased exposure was predicted to decrease the outcome. Instruments were ranked based on the default ranking criterion based on the absolute value of per-allele effect size.

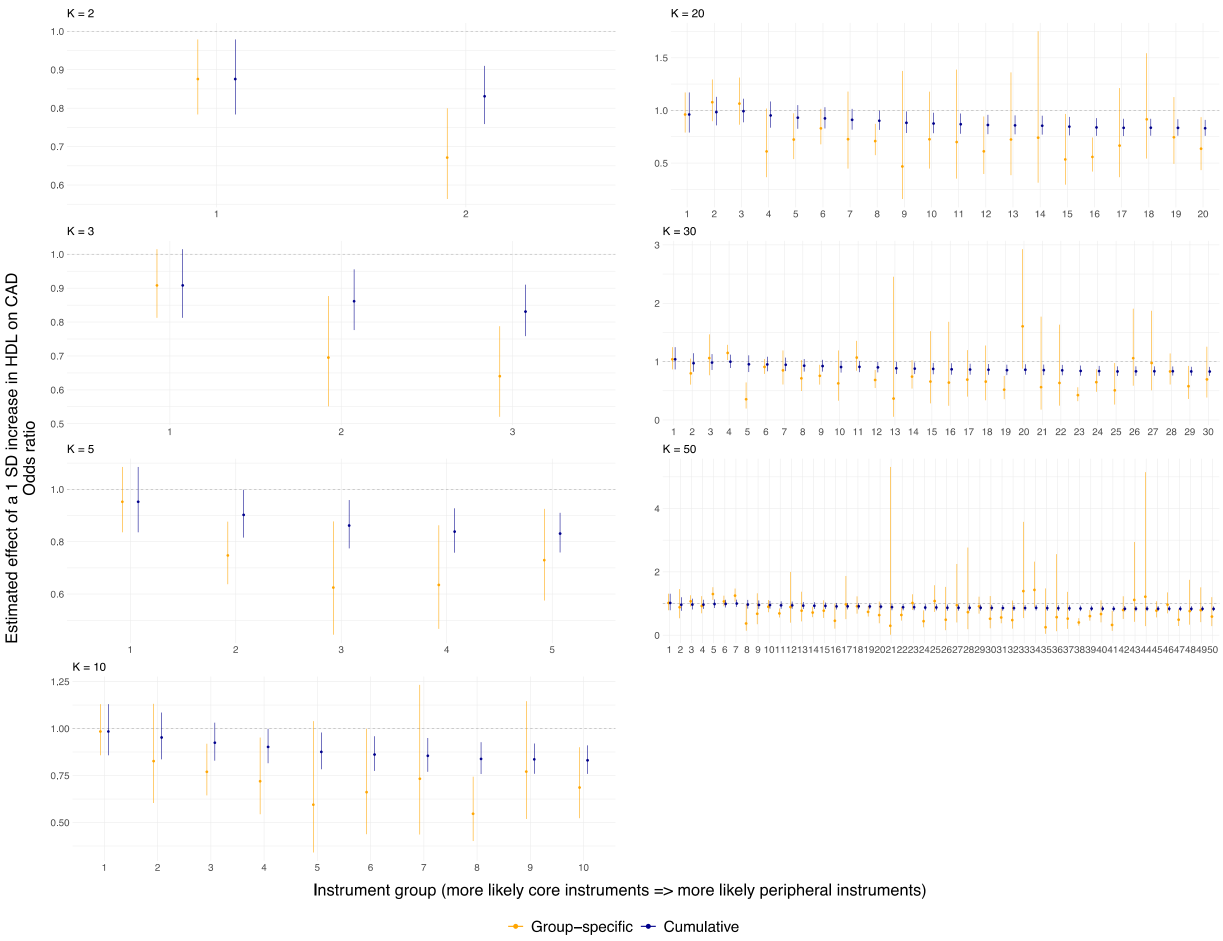

Supplementary Figure S2. Illustration of multiple sensitivity analyses with different values of K. Group-specific and cumulative MR Corge estimates of the effect of serum high-density lipoprotein cholesterol (HDL) levels on the risk of coronary artery disease (CAD) are displayed. Results of these sensitivity analyses consistently show that based on putative core instruments, serum HDL levels may not have a causal effect on the risk of CAD. Estimates obtained using the inverse variance weighted method are illustrated with error bars indicating 95% confidence intervals. SD, standard deviation.

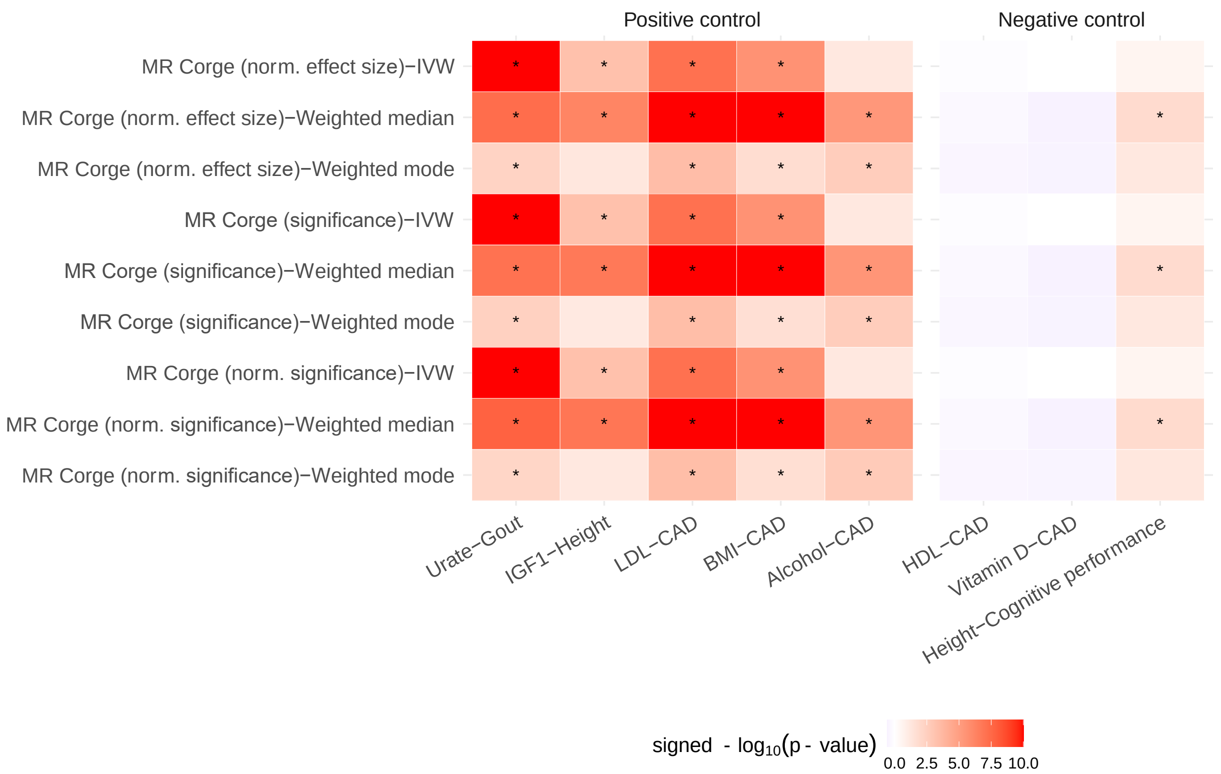

Supplementary Figure S3. Significance of MR Corge estimates in positive and negative controls using alternative instrument ranking criteria. Asterisks (*) indicate estimates with a p-value < 0.05. Red colors represent that increased exposure was predicted to increase the outcome while blue colors represent that increased exposure was predicted to decrease the outcome. P-values $<1.0\text{x}10^{-10}$ were set to $1.0\text{x}10^{-10}$ for visualization purposes.
